# Paroxysmal atrial fibrillation associated with a moderate form of COVID‐19 in a middle‐aged man with low cardiovascular risk factor: More still needs to be done in this topic

**DOI:** 10.1002/ccr3.3923

**Published:** 2021-02-12

**Authors:** Mazou Ngou Temgoua, Sylvain Chanseaume, Enver Hilic, Kane Karamoko, Joel Noutakdie Tochie, Gislain Beyina, Lise Camus, Alexandra Chanseaume, Mischie Alexandru, Khaled Benfreha, Nouhoun Diallo, Romain Eschalier

**Affiliations:** ^1^ Cardiology Unit Center Hospital of Monluçon Monluçon France; ^2^ Department of Internal Medicine and Specialities Faculty of Medicine and Biomedical Sciences Yaoundé Cameroon; ^3^ Department of Anaesthesiology and Critical Care Medicine Faculty of Medicine and Biomedical Sciences Yaoundé Cameroon; ^4^ Department of Cardiology Clermont‐Ferrand University Hospital Clermont‐Ferrand France

**Keywords:** atrial fibrillation, COVID‐19, low cardiovascular risk factor, middle‐aged patient

## Abstract

Strict monitoring of the heart rhythm in patients with COVID‐19 even nonsevere case and patient with low cardiovascular risk factors is very important to prevent fatal outcomes.

## Graphical abstract

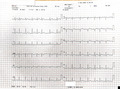

## 1 INTRODUCTION

Cardiac arrhythmia is a major complication of COVID‐19. This occurs often in patients with severe COVID‐19 with pre‐existing cardiovascular diseases. We report an unusual case of paroxysmal atrial fibrillation in an adult male with low cardiovascular risk factor who presented with a moderate form of COVID‐19. A 50‐year‐old patient with uneventful past history, was admitted to the emergency room of the hospital center of Montluçon for irregular palpitations and dyspnoea on physical exertion both of 24 hours duration. Clinical evaluation revealed a non‐ill‐looking patient with mild respiratory distress. A resting electrocardiogram found atrial fibrillation with a rapid ventricular beat response. Biological tests revealed positive COVID‐19 RT‐PCR, a mild hypoxemia (PO_2_ = −65 mm Hg) and a mild inflammatory syndrome (CRP = 34.7 mg/L, range: 0‐5 mg/L and IL‐6 = 30.9 ng/L; range: 0‐7 ng/L). The patient was treated with supplementary oxygen through nasal prongs at 3 liters per min, dexamethasone 6 mg/24 h intravenously (IV), rivaroxaban 20 mg/24 h per os, and verapamil 120 mg/24 h orally. Day 2 after admission was marked by a sinus regular rhythm, which motivated the introduction of Flecainide 100 mg/24 h for normo‐rhythm maintenance therapy. After 2 weeks of hospitalization, the patient returned home with normal clinical and biological parameters. One month later the patient was seen in consultation without any symptoms and he was still on sinus rhythm. Flecainide was, therefore, interrupted since it was the first episode of AF. This atypical case of paroxysmal atrial fibrillation during a nonsevere form of COVID‐19 first alerts clinicians to enhance follow‐up of all COVID‐19 for the earlier detection of rhythm disorders. Another lesson of this case is that more is still to be done for understanding all the cardiovascular implications of the COVID‐19 infection.

Coronavirus disease 2019 (COVID‐19) is a worldwide health crisis responsible for a high cardiovascular burden.^1^ Amongst the cardiovascular disease induced by SARS‐COV2, the viral pathogen of COVID‐19, cardiac arrhythmia is one of the leading causes of mortality.^2^ Wang and colleagues in a retrospective cohort of 138 patients found that cardiac arrhythmia occurs in 16.7% of all COVID‐19 patients and reached 44% in patients admitted in the intensive care unit.^3^ Some hypotheses have been postulated to explain the pathophysiology of arrhythmia during COVID‐19; these include the role of hypoxemia, the induced cytokine storm, and possible direct injury of conductive tissues by the virus.^4^ Ventricular arrhythmia is generally more taken into consideration because of its most lethal effect^5^; In Wuhan, Guo et al found an incidence of 6% of severe ventricular arrhythmia in COVID‐19 patients.^6^ Supraventricular arrhythmia and particularly atrial fibrillation (AF) are the most common arrhythmias associated with COVID‐19 and is potentially responsible for hemodynamic instability and thromboembolic events.^4^ Accounting for 16.5%‐27.5% of the total number of patients according to Colon and colleagues.^7^ The concomitant occurrence of paroxysmal AF and COVID‐19 is generally observed in elderly patients with prior cardiac abnormalities and/or severe inflammatory response.^8^ In this case, the virus and related complications appear like a trigger for rhythm disorder.^8^ Although the scenario of paroxysmal AF in COVID‐19 in patients without major cardiovascular comorbidities is rare. We describe the case of paroxysmal atrial fibrillation in a relatively young patient with low cardiovascular risk factors.

## 2 CASE PRESENTATION

**FIGURE 1:**
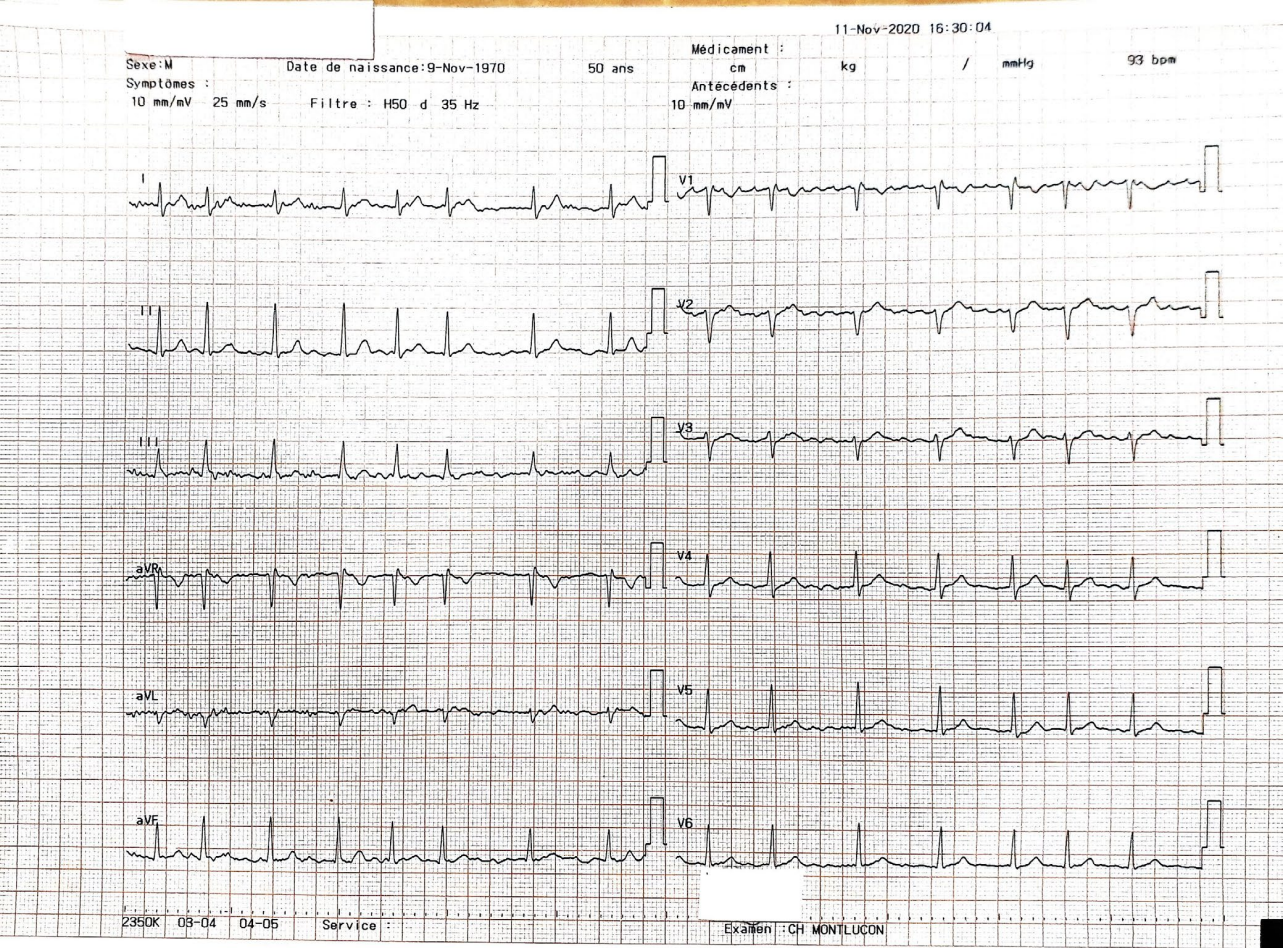
Atrial fibrillation with a ventricular response at 93 bpm

**FIGURE 2:**
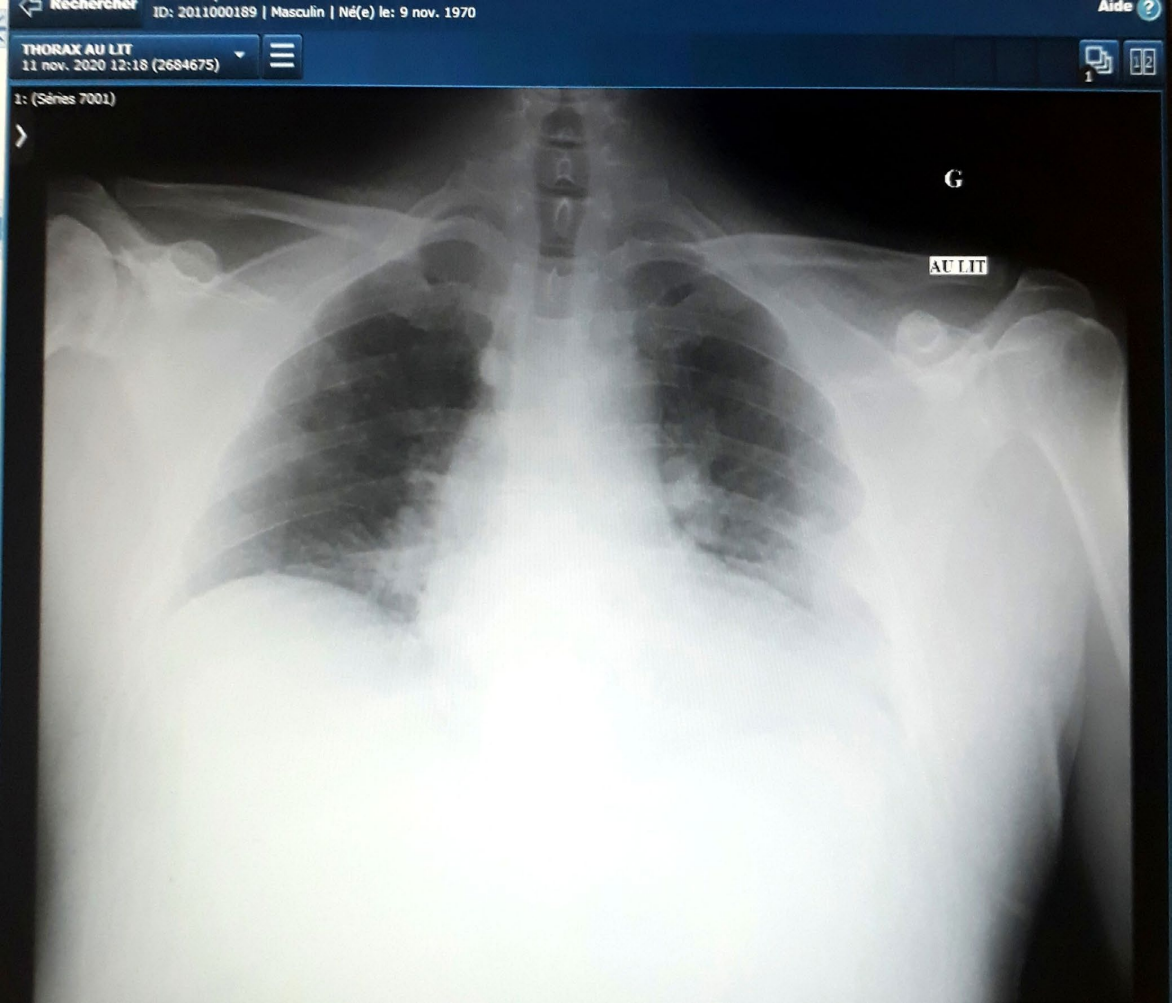
Low transparency of the left basal lung

**FIGURE 3:**
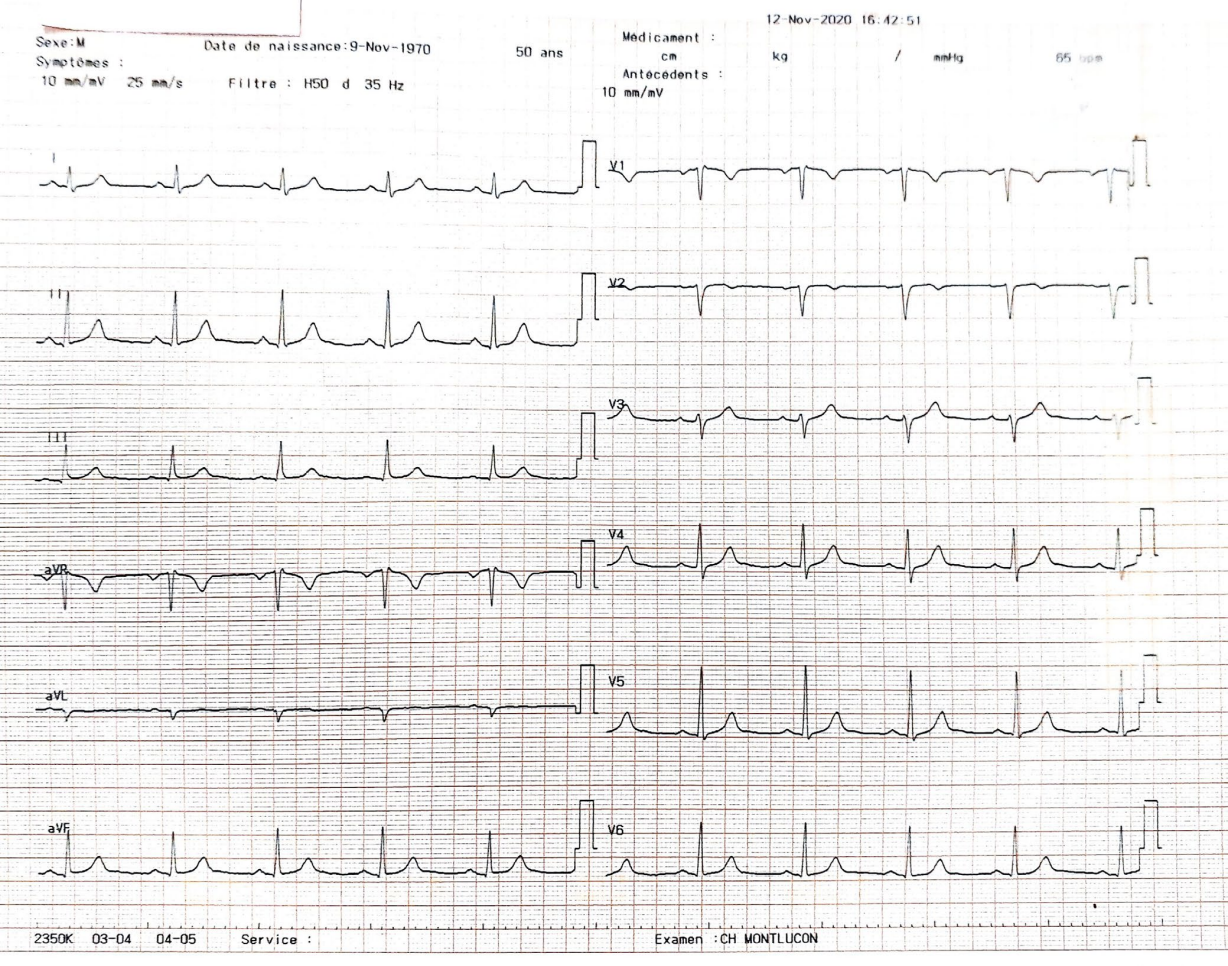
Normal sinus rhythm at day 2 after admission

A 50‐year‐old male engineer was admitted into the emergency room of the hospital center of Montluçon for irregular palpitations and dyspnoea on physical exertion which lasted 24 hours prior to his current presentation. One week ago, he developed anorexia, asthenia, and myalgia. His past medical history was unremarkable apart from asthma for which he took salbutamol spray, budesonide plus formoterol 400/12 mcg twice daily during crises. He had no past history of cardiac arrhythmia, he was nonsmoker, nonalcoholic. He moderately physically exercised. Clinical evaluation revealed a non‐ill‐looking patient with mild respiratory distress (oxygen saturation level = 87%), he was apyretic with a blood pressure of 128/85 mm Hg and a grade I obesity (a body mass index of 31.57 kg/m^2^). A resting electrocardiogram found atrial fibrillation with ventricular beat response (Figure 1). Biological tests revealed a positive RT‐PCR COVID‐19 test, a normal full blood count with: leucocytes of 6100/mm^3^, Hemoglobin level at 17 g/dL and platelet count at 1 72 000/mm^3^, a mild hypoxemia (PO_2_ = −65 mm Hg), elevated NT proBNP at 838 pg/mL (normal range: 0‐125 pg/mL), slightly elevated D‐dimer at 585 mcg/L (normal value <500 mcg/L), and a mild inflammatory syndrome (CRP = 34.7 mg/L, normal range: 0‐5 mg/L and IL‐6 = 30.9 ng/L; normal range: 0‐7 ng/L). Transthoracic echocardiography was normal with a preserved ejection fraction of 60%. Chest X‐ray was unremarkable (Figure 2). The patient received an initial treatment consisting of supplementary oxygen through nasal prongs at 3 liters per min, intravenous dexamethasone 6 mg/24 h for 10 days, rivaroxaban 20 mg/24 h per os, and verapamil 120 mg/24 h per os. Telemetry and daily resting electrocardiogram were used for the monitoring of this arrhythmia. On day 2 after admission, the patient was on sinus regular rhythm with a heart rate at 65 bpm (Figure 3). We, therefore, added Flecainide 100 mg/24 h to the aforementioned treatment for rhythm maintenance therapy. After 2 weeks of hospitalization, the patient returned home with normal clinical and biological parameters. One month later the patient was seen in consultation without any symptoms and he was still on sinus rhythm. Flecainide was, therefore, interrupted since it was the first episode of AF.

## 3 DISCUSSION

Coronavirus disease (COVID‐19) is now considered as a multi‐systemic infectious disease having a great potential to cause severe cardiovascular complications.^9^ Little is known about the occurrence of AF during COVID‐19 infection. Based on the available literature, among COVID‐19 patients, AF was detected in 19% to 21% of all cases.^10^, ^11^ The rate of new‐onset of AF varies between 3.6% and 6.7% according to some clinical studies.^7^ This AF occurs mainly in patients with cardiovascular risk factors and severe respiratory distress.^7^ The pathophysiology of COVID‐19 related AF is not well understood, and hypothesized mechanisms include a reduction in angiotensin‐converting enzyme 2 (ACE2) receptor availability, the inflammatory cytokine storm, the direct viral endothelial damage, electrolytes, and acid‐base balance abnormalities in the acute phase of severe illness and increased adrenergic response to the infection.^13^

New‐onset of AF in COVID‐19 with low cardiovascular disease is exceedingly rare. The only risk factor for AF in our patient was mild obesity. The relationship between obesity and AF is complex and includes atrial inflammation, fibrosis, lipotoxicity, or autonomic dysregulation. These abnormalities are mostly found when obesity is associated with other cardiovascular diseases such as hypertension and diabetes.^12^ Taha and colleagues reported a case of a man of 51 years old with no significant history presented with palpitations, dyspnea on physical exertion, and fatigue. The paraclinical workup of this patient was unremarkable except for left ventricular hypertrophy that oriented the authors to the possibility of a chronic cardiovascular condition.^13^ Seecheran et al reported a second case of a 46‐year‐old Caribbean‐Black man with no relevant past history, clinically this patient had grade 1 hypertension without cardiac involvement and mild respiratory distress syndrome with rapid AF resolved by electric cardioversion.^13^ These cases have three particularity; first, the incidence seems to be higher in the male gender, secondly, the patients have mild to moderate COVID‐19 and thirdly, a low cardiovascular risk factor for all the cases. SARS‐COV2 could be a great arrhythmogenic trigger probably with a male gender correlation. Hence, more studies are warranted to affirm this hypothesis.

## 4 CONCLUSION

The relationship between AF and COVID‐19 is still not clear for the scientific community, this case is a middle‐aged male patient with a low cardiovascular and nonsevere COVID‐19 should alert the scientific community for future research on this topic.

## CONFLICT OF INTEREST

None declared.

## AUTHOR CONTRIBUTIONS

All the authors: Managed the case. MNT: Wrote the manuscript. JNT, SC, EH: Done the critical revision, SC, RE: Supervised.

## ETHICAL APPROVAL

Formal ethical approval from the University Research Ethics Board was not required for the completion of this study.

## INFORMED CONSENT

Written informed consent for publication of this case report was obtained from the patient.

## Data Availability

The data that support the findings of this study are available from the corresponding author upon reasonable request.
